# Atomically Precise Water-Soluble Gold Nanoclusters: Synthesis and Biomedical Application

**DOI:** 10.1021/prechem.3c00036

**Published:** 2023-06-04

**Authors:** Qian Yan, Zhaotong Yuan, Yating Wu, Chunmei Zhou, Yihu Dai, Xiaoyue Wan, Dan Yang, Xu Liu, Nianhua Xue, Yan Zhu, Yanhui Yang

**Affiliations:** † Institute of Advanced Synthesis, School of Chemistry and Molecular Engineering, 91599 Nanjing Tech University, Nanjing 211816, China; ‡ School of Chemistry and Chemical Engineering, Nanjing University, Nanjing 210093, China; § College of Material and Chemical Engineering, Minjiang University, Fuzhou 350108, China

**Keywords:** Gold nanocluster, well-defined structure, water-soluble, biodiagnostic, theranostic

## Abstract

Atomically precise water-soluble gold nanoclusters (Au NCs) protected by organic ligands have attracted growing attention in serving as unique nanomaterials with the potential to generate theranostic tools (bioimaging, biosensing, and biotherapy), due to their ultrasmall size, superior photoluminescence, good biocompatibility, and nontoxicity. More importantly, Au NCs afford a well-defined atomic packing structure and molecular purity, providing a superior platform to unravel the structure–performance correlations for biodistribution, biological pharmacokinetics, and excretion of Au NCs. In this Review, we mainly survey the synthesis of water-soluble Au NCs and the recent progress in biomedicine of Au NCs, including bioimaging, biosensing, and biotherapy. The effects of ligand and size on the biomedical properties are discussed in detail. We hope that the advances in this research area can expand the applications of Au NCs in biomedicine.

## Graphical abstract

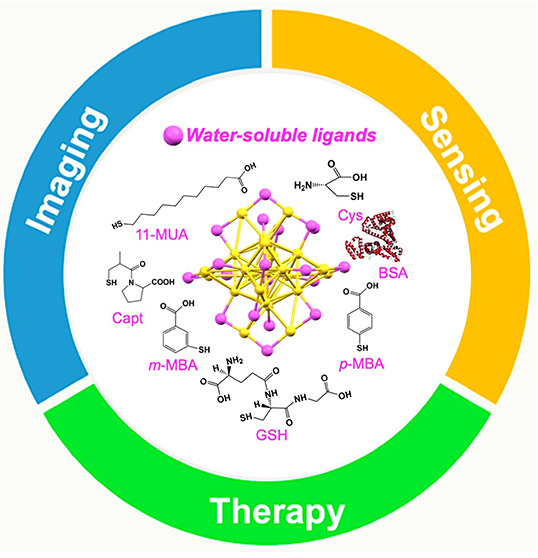

## 1 Introduction

In recent years, nanomaterials and nanotechnology have played a growing role in biodiagnostics and therapeutics.^1,2^ In particular, novel water-soluble gold nanoclusters (Au NCs, Au_*n*_(SR)_*m*_^*q*^, where *n*, *m*, and *q* represent the number of Au atoms, water-soluble ligands SR, and the charge, respectively) with well-defined atomic composition and structure, as unique nanomaterials, have greatly spurred our attention.^3–6^ Compared with the conventional Au nanoparticles (NPs), Au NCs afford core–shell structures consisting of Au(0) core and Au­(I)–S shell (Figure 1d). Au NCs are multinucleated aggregates between Au atoms and Au NPs, which are 1–3 nm.^7^ In this size range, strong quantum confinement effects make Au NCs exhibit discrete energy levels like that of the Au atom (Figure 1a), showing a unique UV–vis absorption caused by the HOMO–LUMO transition (Figure 1b,c) and photoluminescence (PL, Figure 1d,e) properties.^8,9^ Note that the PL mechanism of Au NCs is different from the large Au NPs with continuous energy levels due to the diverse interactions between Au NCs and light. As shown in Figure 1e, Au NCs have two unique pathways of charge transfer between the HOMO and LUMO levels: (1) ligands to the core metal charge transfer (LMCT) via Au–S interaction; and (2) ligands to core metal and motif metal charge transfer (LMMCT), determining the PL properties of Au NCs. The Au cores and ligands can affect the degree of LMCT and the LMMCT due to the different electron withdrawing of Au cores and the electron-donating of ligands. We take Au_25_ as an example to clarify the PL mechanism. The PL of Au_25_ consisted of visible light (Vis) and near-infrared (NIR) emissions, which was due to the electronic transition between HOMO and LUMO. The pathways of the Vis and NIR emissions were remarkably different. The former had a rapid decay with a lifetime of less 1 ps, which resulted from the core Au atoms. Whereas the latter had a long lifetime, which was related to the motif staple of the Au_25_ NC (i.e., 6 kinds of −S–Au–S–Au–S−). In terms of optical and physicochemical properties of Au NCs, it is very suitable for biomedical applications, which it is not only because of the ultrasmall size (favor renal excretion) and the increased PL (favor detection in *vivo* via multimodal imaging techniques), but also because of the high stability in serum and urine, high biocompatibility, and low toxicity.^10^ Meanwhile, Au NCs can provide an ideal platform to understand the mechanism to regulate fluorescence performance via designing the NC structure, and reveal structure–properties relationships on the basis of the biodistribution, biological pharmacokinetics, and elimination of Au NCs in vivo.^11–15^

**Figure 1:**
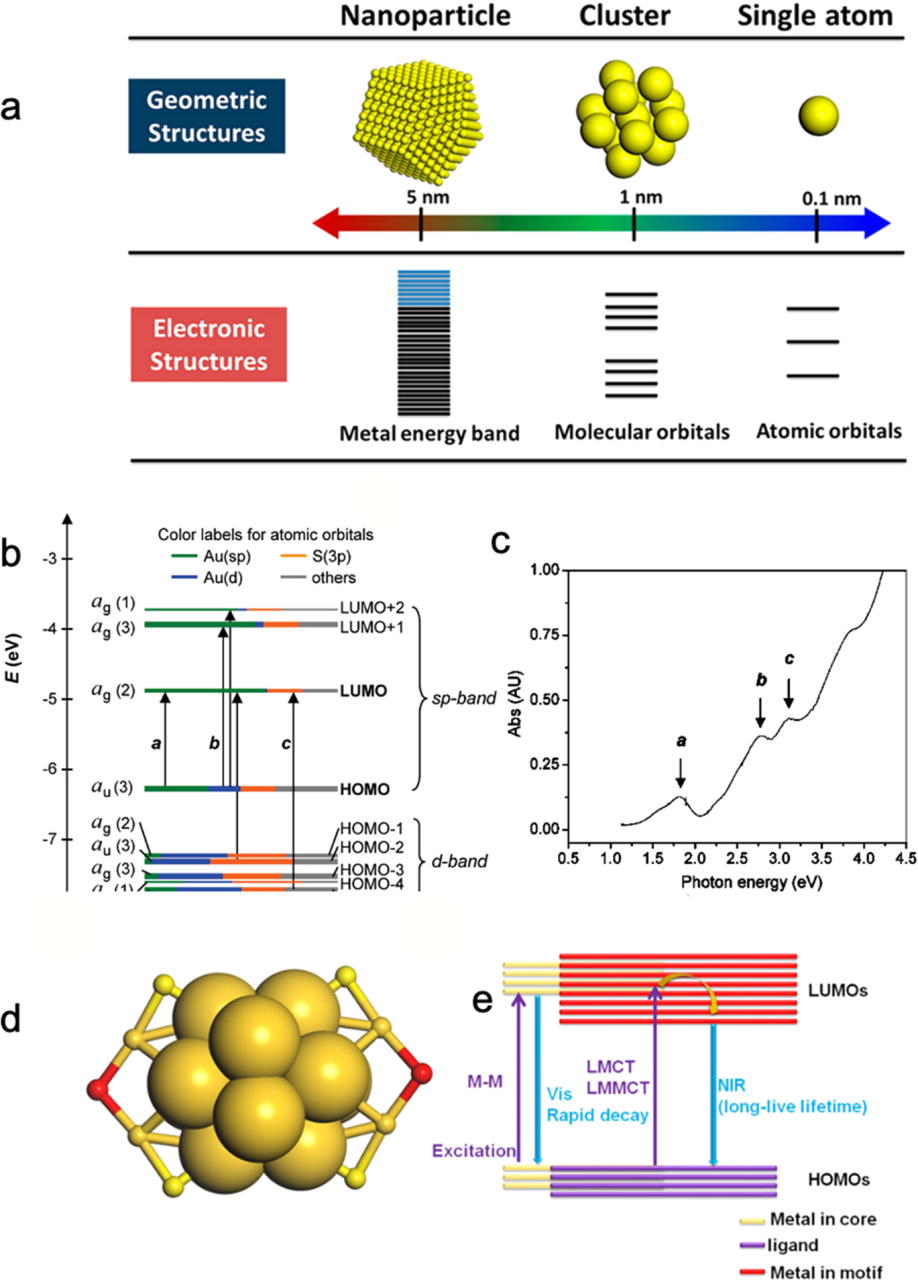
(a) Structures and sizes of single atoms, NCs, and NPs. (b) K–S orbital energy level diagram and (c) UV–vis spectra of [Au_25_(SR)_18_]^−^. (d) Structure of [Au_25_(SR)_18_]^−^. (e) Schematic illustration of the photoluminescence mechanism of [Au_25_(SR)_18_]^−^. (a) Reproduced from ref 7. Copyright 2018 American Chemical Society. (b,c) Reproduced from ref 8. Copyright 2008 American Chemical Society. (d,e) Reproduced from ref 9. Copyright 2021 Elsevier Ltd.

To date, researchers have made significant advances toward hydrophobic Au NCs with their preparation, crystal analysis, and structure–properties correlations of catalysis, and the research progress has been surveyed.^16–20^ In addition, there are several reviews on water-soluble metal NCs, mainly focusing on the bio-NCs, a new type of biomolecule-metal NC composites.^21–23^ Namely, they highlight the important roles of biomolecules (e.g., proteins, peptides, and DNA) in water-soluble metal NCs synthesis, as well as the effects of metal NCs on the structures and properties of biomolecules. In addition, there are a few reviews that summarize the luminescence properties of water-soluble metal NCs.^24,25^ Unfortunately, some of these bio-NCs do not have molecular purity or a well-defined number of metal atoms. In this Review, we highlight the synthesis of atomically precise water-soluble Au NCs protected by different organic ligands including glutathione (SG), captopril (Capt), 1-mercaptoundecanoic acid (1-MUA), 3-mercaptobenzoic acid (*m*-MBA), 4-mercaptobenzoic acid (*p*-MBA), 6-mercaptohexanoic acid (MHA), cysteine (Cys), and bovine serum albumin (BSA), and the research advances in biodiagnostic and therapeutic applications, including imaging, sensing, and therapy. In particular, the influence of organic ligands and core sizes on the biomedical properties is discussed in detail. At last, we present the challenges and opportunities for broadening the biomedical applications of Au NCs.

## 2 Synthesis of Water-Soluble Au NCs

For exploring the physicochemical properties of water-soluble Au NCs and boosting their wider applications in biomedicine, it is of significance to prepare water-soluble Au NCs with high purity and yield. Therefore, researchers have made extensive efforts toward the synthesis and separation of water-soluble Au NCs.

In early reported work, the Whetten group prepared a series of Au NCs protected by GSH with 4.3, 5.6, 8.2, and 10.4 kDa molecular weights (MW).^26,27^ They separated the Au NCs with MW of 10.4 kDa via polyacrylamide gel electrophoresis (PAGE) and initially inaccurately identified the composition as Au_28_(SG)_16_.^28,29^ Although controlled synthesis at the atomic level was not available, their synthetic work provided a foundation for the synthesis of ultrasmall Au NCs. For example, utilizing large amounts of excess thiols (Au-to-thiol mole ratios of 1:3 or higher) to transform Au (III) to Au (I)-SR; utilizing great amounts of excess reductant (e.g., Au-to-NaBH_4_ mole ratios of 1:10) to reduce Au (I) to Au (0).^30^

Subsequently, the Tsukuda group improved the PAGE separation technique and, for the first time, prepared and separated numerous relatively pure SG-capped Au NCs, including Au_10_(SG)_10_, Au_11_(SG)_11_, Au_12_(SG)_12_, Au_15_(SG)_13_, Au_18_(SG)_14_, Au_22_(SG)_16_, Au_22_(SG)_17_, Au_25_(SG)_18_, Au_29_(SG)_20_, Au_33_(SG)_22_, Au_35_(SG)_22_, Au_38_(SG)_24_, and Au_39_(SG)_24_ (Figure 2).^31^ Of note, they found the composition of Au_28_(SG)_16_ reported by the Whetten group was actually Au_25_(SG)_18_. Later Au_25_(SG)_18_ was believed to afford a crystal structure analogous to that of hydrophobic Au_25_(SR)_18_.^32^ Unfortunately, the poor purity was the major problem for synthesizing water-soluble Au NCs because it was hard to separate from the Au_*n*_(SG)_*m*_ mixtures via PAGE.^26–29,31,33^ Thus, the high purity and large-scale synthesis of Au NCs remained challenging at that time.

**Figure 2:**
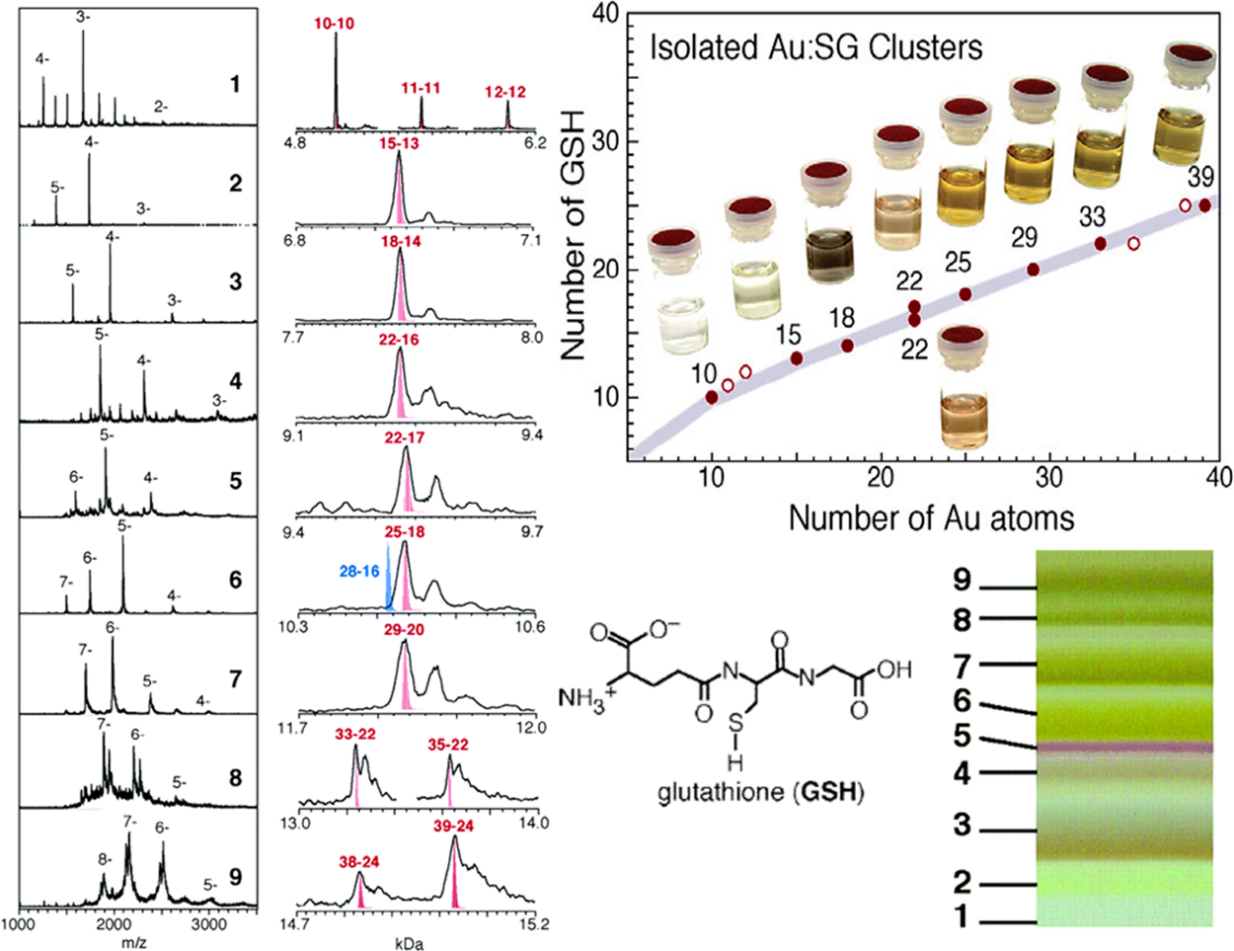
PAGE results of the mixture Au_*n*_(SG)_*m*_ NCs and the corresponding low-resolution ESI MS. Reproduced from ref 31. Copyright 2005 American Chemical Society.

In 2005, the Tsukuda group prepared Au_25_(SG)_18_ utilizing Au_11_ capped with triphenylphosphine (PPh_3_) at the large-scale level (70 mg) through the ligand exchange method.^34^ In 2014, the Hutchison group prepared and separated two kinds of triphenylphosphine-stabilized Au_11_, including [Au_11_(PPh_3_)_8_Cl_2_]Cl and Au_11_(PPh_3_)_7_Cl_3_ NCs (Figure 3).^35^ Surprisingly, they found that it yielded only small NCs from [Au_11_(PPh_3_)_8_Cl_2_]Cl through a ligand exchange method, whereas it yielded Au_25_(SG)_18_ from Au_11_(PPh_3_)_7_Cl_3_ (Figure 3). The results confirmed that the reactivity of Au_11_ NCs may be closely related to its ligand shell, which largely determined the stability of Au_11_ NCs. Specifically, [Au_11_(PPh_3_)_8_Cl_2_]Cl with la arger amount of PPh_3_ was more stable and less likely to react with GSH to form the larger Au_25_ NCs. Au_11_(PPh_3_)_7_Cl_3_ with less dense PPh_3_ was less stable and easier to react with SG to form Au_25_ NCs. In addition, the Pradeep group observed the reactions of Au_25_(SG)_18_ with other ligands including 3-mercapto-2-butanol (MB), *N*-acetyl-glutathione (NAGSH), and *N*-formyl-glutathione (NFGSH) to prepare Au_25_-MB, Au_25_-SGAN, and Au_25_-SGFN, respectively, via a ligand exchange method.^36^

**Figure 3:**
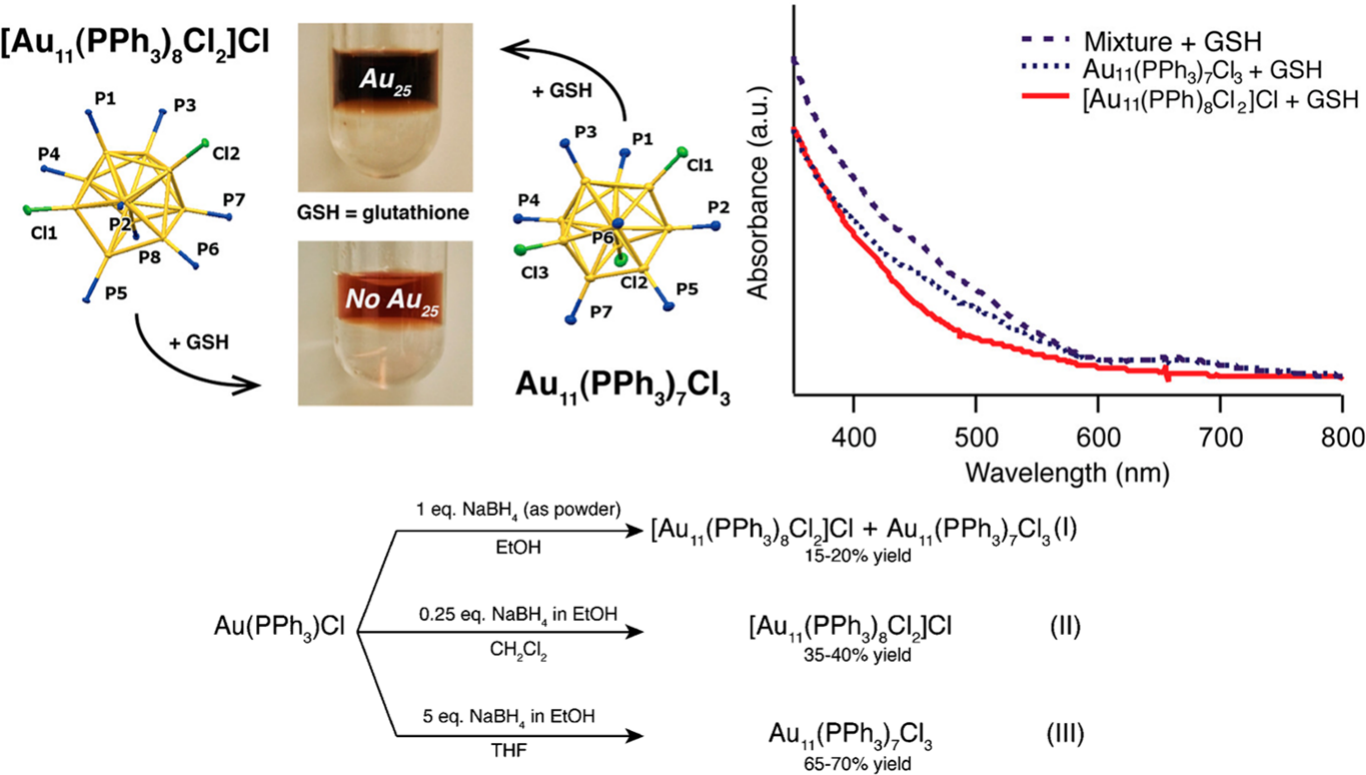
Preparation of triphenylphosphine-stabilized Au_11_ NCs. Reproduced from ref 35. Copyright 2014 American Chemical Society.

Aside from the ligand exchange method, the Xie group reported a new strategy to synthesize water-soluble Au NCs, namely, using a mild CO reductant instead of NaBH_4_ and changing the pH value of the reaction solution to slow down the reaction rate to synthesize a series of Au NCs. As shown in Figure 4, they prepared Au_10–12_(SG)_10–12_, Au_15_(SG)_13_, Au_18_(SG)_14_, and Au_25_(SG)_18_ NCs via adopting a CO reducing agent and regulating pH values to be 7, 9, 10, and 11, respectively.^37^ Then, they synthesized several different ligand-stabilized water-soluble Au_25_ NCs at the gram-scale through a NaOH-mediated NaBH_4_ reduction method, especially for Au_25_ capped by two or three types of thiolates.^38^ Of note, sodium hydroxide was utilized to adjust the reaction kinetics by slowing down the reduction rate of NaBH_4_ and increasing the etching rate of thiols.

**Figure 4:**
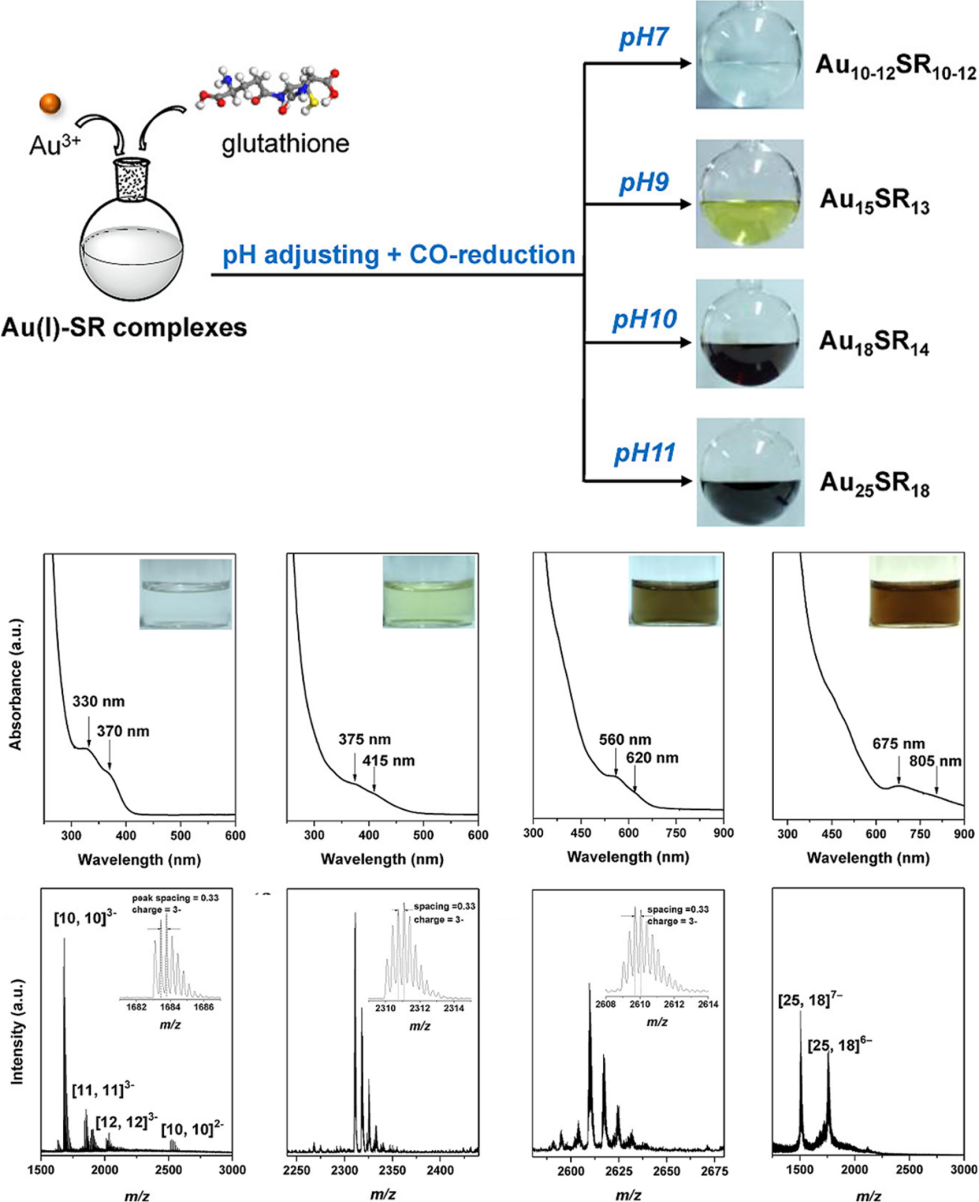
Synthesis of a series of water-soluble Au NCs via using a CO reductant and tuning the pH, as well as the UV–vis and mass spectra. Reproduced from ref 37. Copyright 2013 American Chemical Society.

In addition, Xie’s group also reported a protection–deprotection method for synthesizing Au_25_(Cys)_18_, in which the cetyltrimethylammonium bromide (CTAB) surfactant was used to protect the Cys-Au­(I) species and then removed by dissolving in toluene.^39^ This method could rapidly synthesize Au_25_(Cys)_18_ within 10 min (Figure 5). Note that a certain amount of NaOH was necessary for synthesizing Au_25_(Cys)_18_. It was due to the enhanced electrostatic interaction between CTAB and Cys, which could improve to form the protecting CTAB layer and in turn boost the formation of Au_25_(Cys)_18_ inside CTAB layer. In addition, several water-soluble Au_25_ NCs capped by other ligands have been reported, including BSA and Capt ligand-stabilized Au_25_.^40,41^ Interestingly, Au_25_(Capt)_18_ showed superior thermal stability in contrast to Au_25_(SG)_18_ due to the enhanced stability of the captopril ligand.^41^

**Figure 5:**
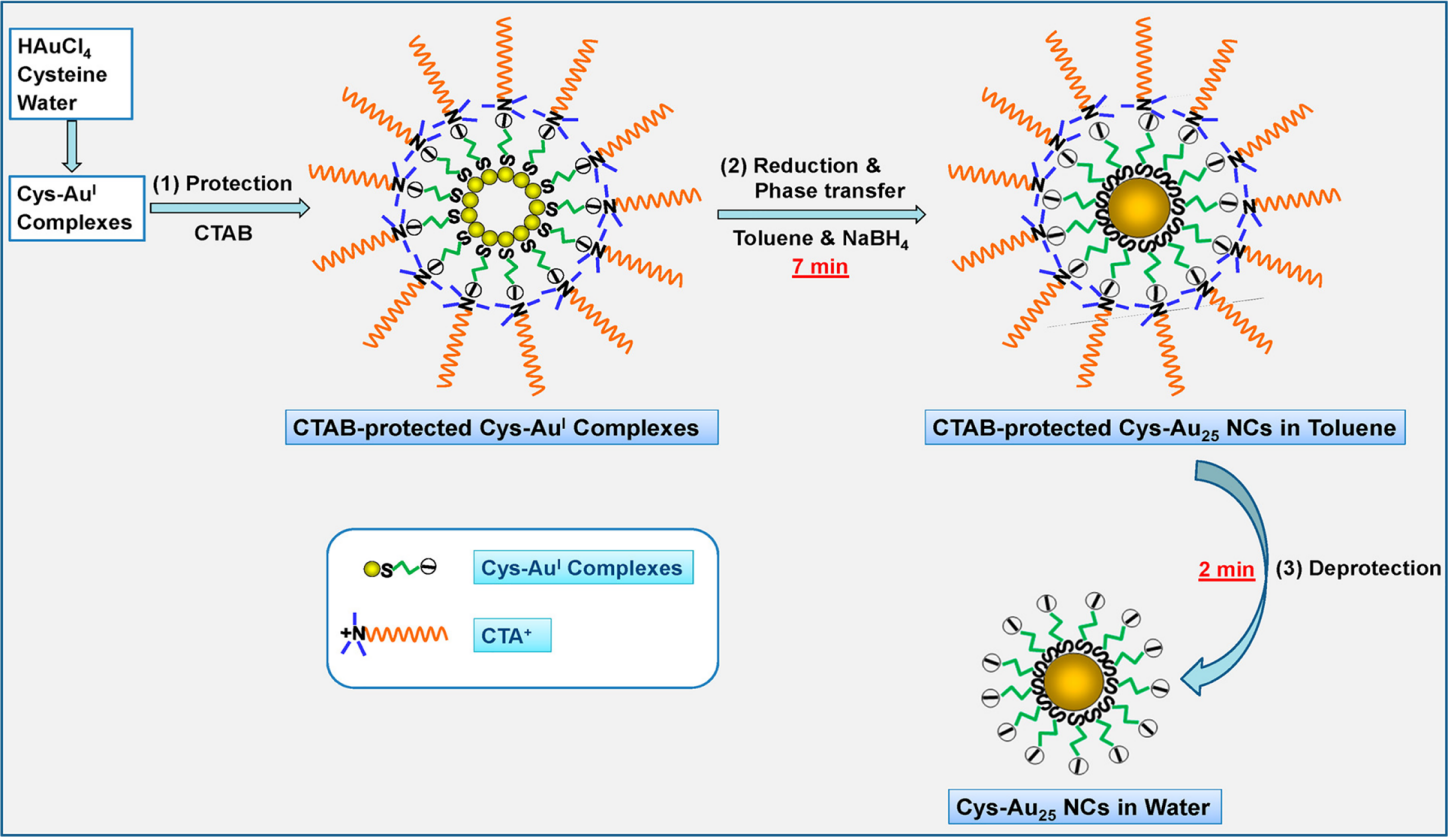
Synthesis of Au_25_(Cys)_18_ via a protection–deprotection method. Reproduced from ref 39. Copyright 2012 American Chemical Society.

Among these water-soluble Au NCs, Au_25_ protected by different water-soluble ligands including GSH,^31^ Cys,^39^ BSA,^40^ Capt,^41^ 11-MUA,^42^ *m*-MBA,^43^ and *p*-MBA^44^ takes a very special place due to the early discovery, easier preparation, and high stability. Table 1 presents the synthetic methods of water-soluble Au_25_, such as the one-pot method, ligand exchange method, CO-reduction method, NaOH-mediated NaBH_4_ reduction method, and protection–deprotection method. It shows that most of the syntheses of water-soluble Au NCs could be achieved at a large scale. Meanwhile, it is revealed that almost all of these reactions were performed under strong alkaline conditions. On the one hand, utilizing alkali conditions could decrease the reduction rate of the strong reductant NaBH_4_ and enhance the etching rate of thiols during the reaction process; on the other hand, the alkaline conditions could boost the electronic interaction between the additive molecule and ligand to facilitate the formation of Au NCs. Furthermore, partial reactions required heating and specific solvent. It is revealed that several parameters exist, such as the type and concentration of ligand and reductant, reductant strength, solution pH values, and reaction temperatures, that affect the synthesis process. Thus, we need to employ deep learning methods to accelerate the synthetic process.

**Table 1:** Synthetic Methods of Water-Soluble Au_25_ NC

synthetic method	reaction conditions	Au NC	scale	reference
one-pot method	water phase, ∼0 °C, < 2 h	Au_*n*_(SG)_*m*_ mixture	small	^26–33^
ligand exchange method	water and chloroform phases, 35 °C, 55 °C, 3 h	Au_11_^+^ → Au_25_(SG)_18_	70 mg	^34^,^35^
CO-reduction method	NaOH, pH = 11, 24 h	Au_25_(SG)_18_	>1 g	^37^
NaOH-mediated NaBH_4_ reduction method	water phase, rt, 1 M NaOH, <3 h	mixed thiols with carboxyl, amine, and hydroxy groups	>1 g	^38^
protection–deprotection method	water–toluene phase, rt, 1 M NaOH, CTAB, <10 min	Au_25_(Cys)_18_	500 mL reaction solution	^39^

Machine learning is a powerful tool in optimizing the synthesis parameters and guiding the experimental designs, which can help us to synthesize more water-soluble Au NCs in a time-saving manner. Recently, the Wang and Xie group adopted machine learning to optimize the synthesis parameters of Au_25_ NC, mainly including temperature, pH, the concentrations of ligand, HAuCl_4_, and reducing agent.^45^ The results are shown in Figure 6 and reveal that (1) the aliphatic ligands were more likely to synthesize Au NCs in contrast to use the aromatic ligands; (2) the molar ratio of ligand-to-Au was <6; (3) the reaction temperature should be below 50 °C when the concentration of reducing agent is below 52 nM; (4) the pH value must be below 12.8 when the concentration of reducing agent is above 52 nM. Significantly, it was a meaningful start to bridging machine learning with metal NCs synthesis.

**Figure 6:**
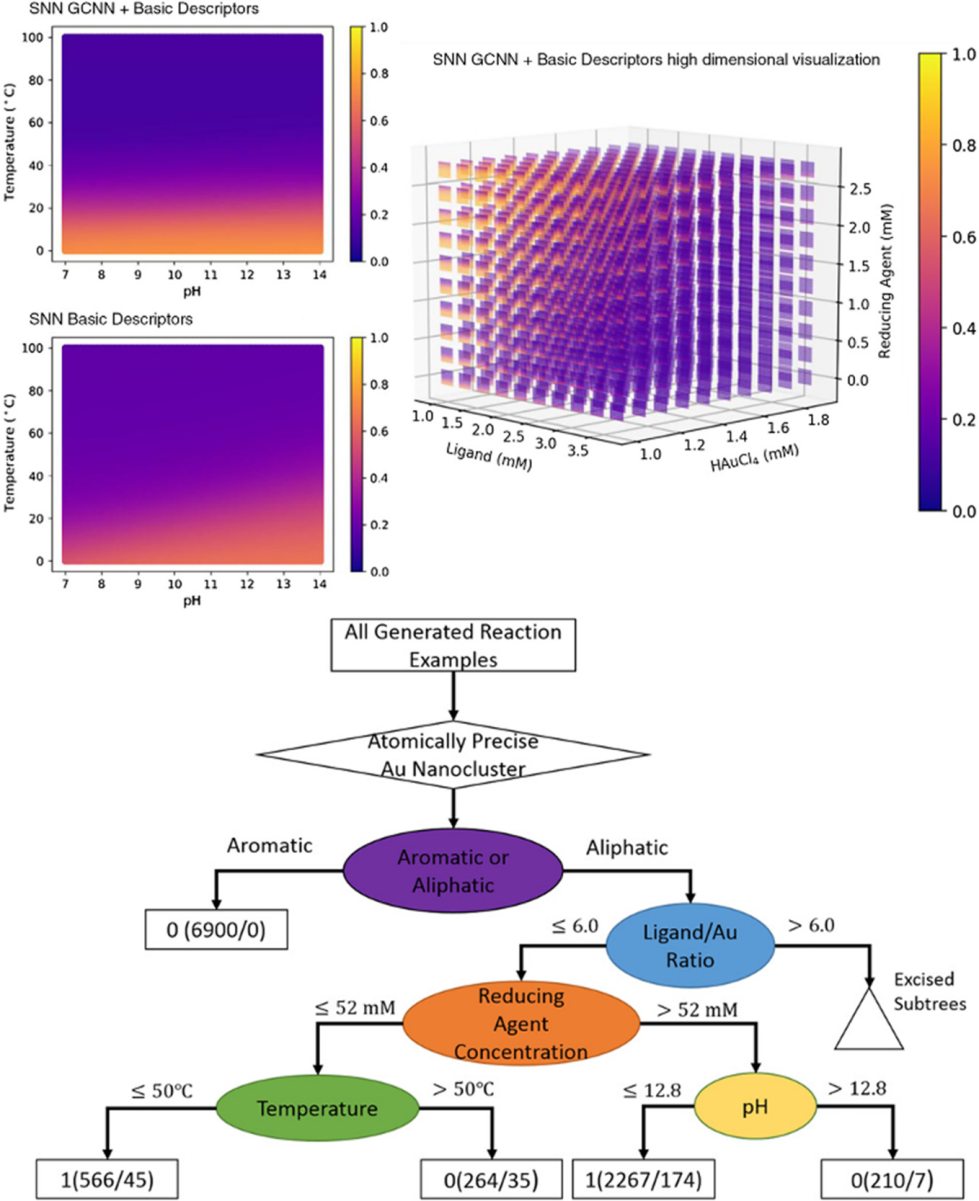
Utilization of machine learning to optimize the synthesis parameters and guide the experimental designs to boost the synthesis of more water-soluble Au NCs. Reproduced from ref 45. Copyright 2019 John Wiley & Sons, Inc.

## 3 Biological Applications

Water-soluble Au NCs have been widely used in biomedical research for the following reasons: first, they are nontoxic and possess good biocompatibility and photostability; second, the small size makes Au NCs easily enter the cell; third, the enhanced luminescence could easily label the cell; fourth, the functional ligands allow Au NCs to have the potential to be drug delivery carriers; last, Au NCs show potential for anticancer application. In this section, we mainly review the recent advances of water-soluble Au NCs in bioimaging, biosensing, and biotherapy.

### 3.1 Bioimaging and Biosensing

Generally, the bioimaging process at the cellular, subcellular, and molecular levels in real-time is conducive to the early detection and treatment of deadly diseases such as cancer.^46,47^ In particular, PL bioimaging has drawn much attention due to the development of nanomaterials with high PL properties.

Some studies suggested that the Au NCs PL intensity was closely associated with the surface ligands. The Wu group explored the ligand role in Au NCs PL, suggesting three kinds of ways to increase the Au_25_ PL properties:^48^ namely, (1) increasing the ligand electronegativity to donate more electrons; (2) increasing the Au core electropositivity; (3) using ligands with electron-rich atoms, such as N and O atoms. As reported, the quantum yield (QY) of water-soluble Au_25_ NCs was ∼2 orders of magnitude higher than that of the oil-soluble Au_25_ NCs.^48^ In 2009, the Xie group reported that BSA-protected Au_25_ NCs had an excellent QY of 6% at 640 nm.^40^ Meanwhile, they prepared a series of BSA-protected Au_4_, Au_8_, Au_10_, Au_13_, and Au_25_ NCs via modulating protein conformation, in which BSA-Au_25_ presented a longer fluorescence lifetime and thus led to polarization.^49^ Thus, it could be studied for time-gated detection in microscopy and imaging. In 2011, the Liu group explored the fluorescence resonance energy transfer (FRET) between POSSFF and the BSA-Au_25_ NCs in vitro in cells, revealing that the FRET system was effective for multicolor intracellular sensing of Hg^2+^ (Figure 7).^50^ Interestingly, the precise action sites as well as the absorption and desorption process of biomaterials can be obtained based on bioimaging in vivo or in vitro.

**Figure 7:**
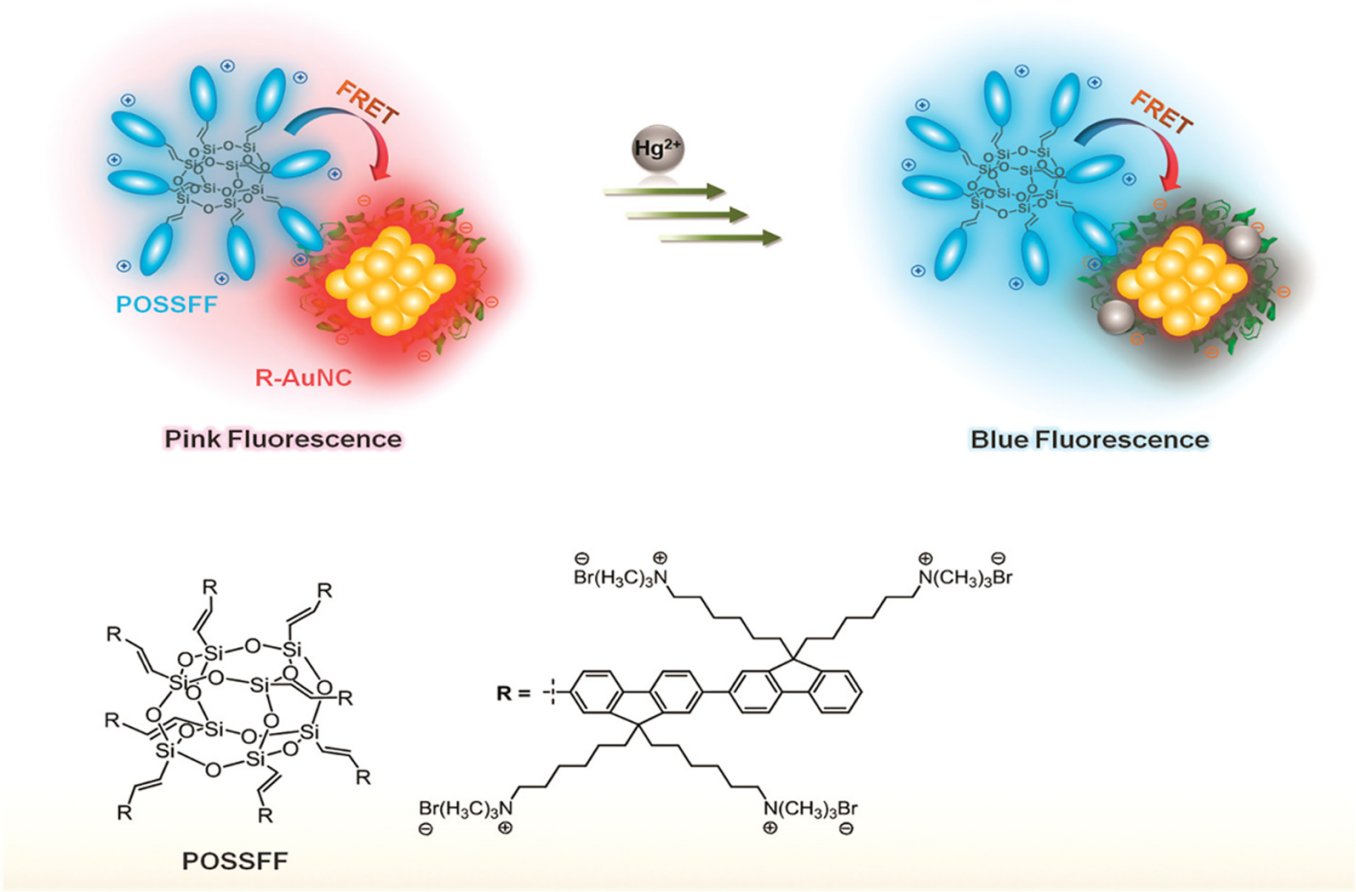
Scheme of visual detection of Hg^+^ based on FRET. Reproduced from ref 50. Copyright 2011 American Chemical Society.

In 2013, Ackerson et al. explored the structure–property correlation for the absorption, biodistribution, metabolism, excretion (ABME), and pharmacokinetics (PK) of Au_25_(SG)_18_, Au_102_(*p-*MBA)_44_, and oligoethylene glycol modified Au NCs in a murine model through bioimaging and biolabeling in vivo.^15^ They reported that the ABME mechanism and blood PK depended not only on the hydrodynamic diameter, but also on the naked hydrophobic surfaces and the surface charge density. Furthermore, they revealed that the renal clearance, transient lung cumulation, and reticuloendothelial system cumulation depended on the Au NCs surface structures.

The second near-infrared (NIR-II, 1100–1700 nm) bioimaging shows excellent potential applications in vascular related medical diagnoses because of the clarity and deep penetration in biological tissues, leading to reducing the tissue scattering and its fluorescence with increasing wavelengths.^51^ However, most of the NIR-II fluorophores exhibits slow excretion and low brightness, limiting their biomedical application. Recently, the Zhang and Xie group revealed that the fluorescence bioimaging depth of Au_25_(SG)_18_ was remarkably enhanced through modulating the PL window to the NIR-II/shortwave infrared (SWIR) window.^52^ The emission of Au_25_(SG)_18_ located at 1120 nm ensured the long wavelength photon to cross the biological barrier and penetrate the deep biological tissues, significantly increasing the spatial resolution. The Au_25_(SG)_18_ NC mediated NIR-II imaging was used to identify the accurate locations of damaged blood vessels in brain tissues. The results showed that there exists higher uptake for Au_25_(SG)_18_ NCs in tumor induced regions of the mice compared with normal tissues, which could clearly identify the primary tumor, vessel, and metastases.

### 3.2 Biotherapy

In addition to their application in diagnostics, such as bioimaging, water-soluble Au NCs can be utilized as therapeutic agents. Promoting the photodynamic and photothermal therapy for cancer treatments is the current trend.

Generally speaking, producing highly reactive singlet oxygen ^1^O_2_ is essential for cancer treatment via utilizing photodynamic therapy. In 2014, the Jin group reported that the Au_25_(Capt)_18_ showed superior biocompatibility and photodynamic activity against the cancer cell.^53^ The results demonstrated that the singlet oxygen ^1^O_2_ could be effectively performed via directly photosensitization for Au_25_(Capt)_18_ NC under NIR photoexcitation, which was important for the Au NCs in the photodynamic applications. In 2018, the Zhu group revealed that the Au_18_(SG)_14_ was accumulated at the lysosomes sites, while Au_18_(SG)_12_(MTPB)_2_ (MPTB = (4-mercaptobutyl) triphenylphosphonium bromide) was more intend to concentrate in the mitochondrial sites to obtain the target switching.^54^ Likewise, it showed that ^1^O_2_ was formed by these two Au_18_ NCs under the photoexcitation at 638 nm. Moreover, it was demonstrated that the Au_18_(SG)_12_(MTPB)_2_ with less amount of ^1^O_2_ exhibited the same biotoxicity for the cancer cells compared with the Au_18_(SG)_14_, which indicated superior photodynamic activity in mitochondria sites on killing the cancer cells than that in the lysosomal sites. Moreover, the Li group explored photothermal therapy of the Au_25_(SG)_18_ for the MDA-MB-231 breast cancer cells, which showed good photothermal activity in attaining 100% cancer cells death under the laser excitation.^55^ This new discovery might expand the applications of Au NCs in the photothermal therapy for the major illnesses.

In addition, the Xie group reported two kinds of new radiosensitizers including Au_25_(SG)_18_ and BSA-Au_25_ NCs for cancer radiotherapy, in which Au_25_(SG)_18_ was intended to accumulate in the tumors and in turn exhibit more excellent enhancement for cancer treatment compared with BSA-Au_25_ and large Au NPs.^56^ The remarkable decrease of the tumor was attributed to the damage of DNA resulting from the photoelectric effect of Au_25_ NCs. Notably, Au NCs could be used as radiosensitizers because they inherited the intriguing characteristics of both the Au cores and the Au–S shells. The former had strong radiotherapy enhancement, and the latter showed good biocompatibility from the SG or BSA ligand. In addition, they revealed that the kidney could efficiently clear Au_25_(SG)_18_ and therefore minimize any secondary action for the body, which might promising as biotherapeutic drugs. Unfortunately, larger BSA-Au_25_ NCs were not efficiently cleared, which might lead to liver damage. Therefore, the size and structure of the coating ligand had a significant effect on the cancer radiotherapy performance.

Furthermore, the Zhang and Xie group first explored the potential of Au_10–12_(SG)_10–12_ as radiosensitizers for consuming tumors and for cancer radiotherapy in mice grafted with a human cervical U14 tumor.^14^ It was revealed that the tumor size was not decreased in mice treated with only Au NCs, whereas the tumor size reduced by 8% under radiation and 65% with combined Au_10–12_ NCs and radiation. They proved that the increased consuming tumor and targeting cell specificity were due to the enhanced EPR (enhanced permeability and retention) effect; namely, the glutathione shell boosted the uptake of tumor by inhibiting the absorption of Au NCs and motivating the transporter on the cell surface. The same experiment was performed by using Au_29–43_(SG)_27–37_, revealing that the size of the tumor in mice treated by only irradiation and both Au NCs and irradiation decreased by 10% and 76%, respectively.^57^

In addition, the Xie group studied the antimicrobial activity of Au NPs and Au_25_(MHA)_18_ NC.^58^ They showed that Au_25_(MHA)_18_ afforded stronger antimicrobial activity in contrast to the large Au NPs (Figure 8). Namely, Au_25_(MHA)_18_ could kill both Gram-positive and Gram-negative bacteria, which was because of the strong interaction between the ultrasmall Au NCs and the bacteria. And thus, it caused bacterial metabolic imbalance, leading to the production of more active oxygen to kill the bacteria.

**Figure 8:**
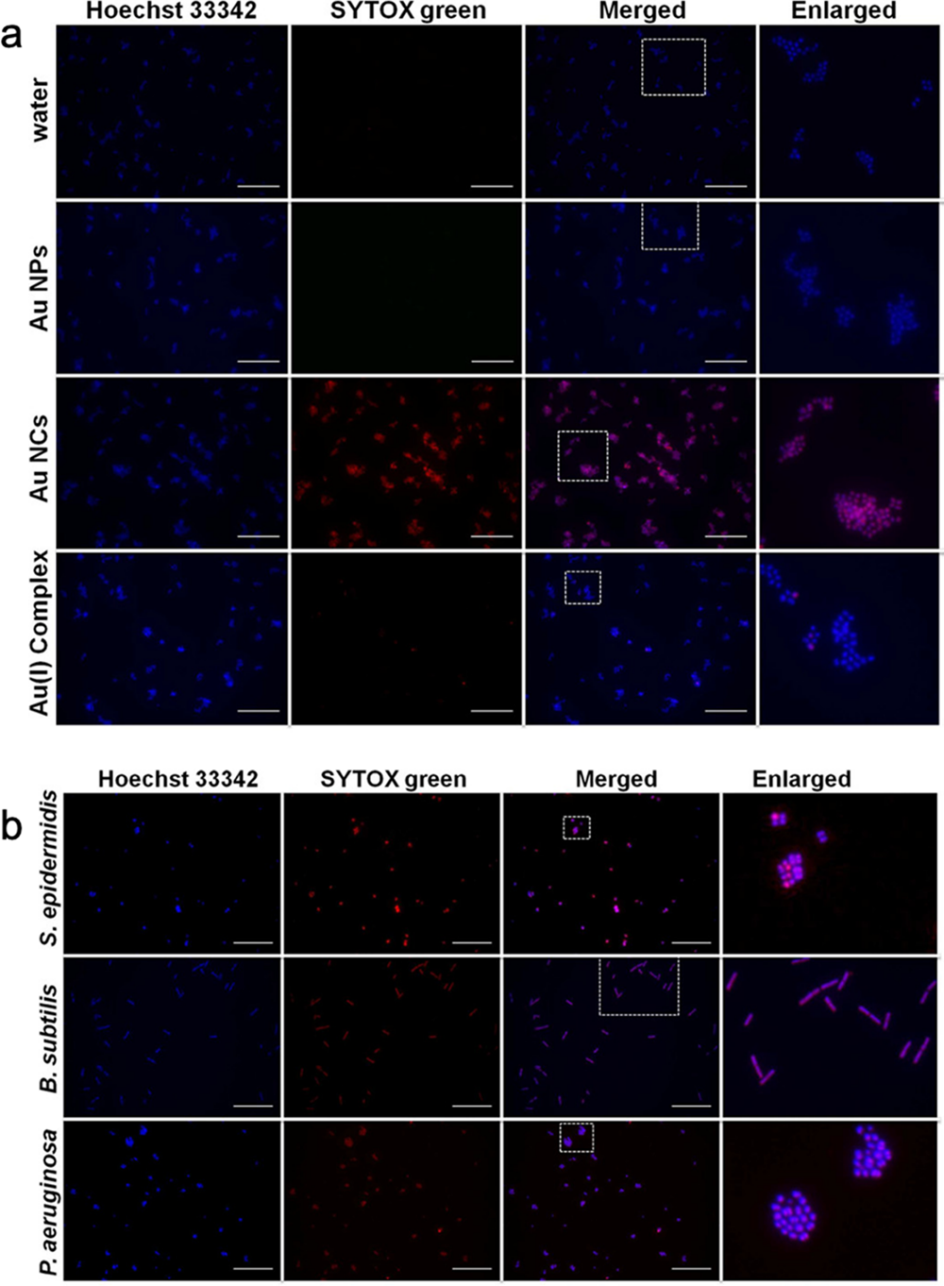
Antimicrobial activity of Au_25_(MHA)_18_ in contrast to the large Au NPs toward (a) *Staphylococcus aureus* bacteria and (b) other bacteria. Reproduced from ref 58. Copyright 2017 American Chemical Society.

As we know, the glomerular filtration barrier can retain the NPs with a size of 6–8 nm and the kidneys can rapidly excrete smaller NPs.^59^ Surprisingly, the Zheng and Xie group found different glomerulus behaviors for Au NCs with a subnanometer size; that is, the renal clearance increased exponentially with the increased quantity of Au atoms among the Au_10–11_, Au_15_, Au_18_, and Au_25_ NCs (Figure 9).^60^ The novel inverse pore size-dependent filtration was due to the Au NCs being more likely to be retained by the endothelial glycocalyx of the glomerulus; specifically, the smaller the NCs, the more easily they are retained. In addition, they showed that the unique NCs-bio interactions in vivo reduced the Au NC spillover from the normal blood vessels and increased the passive targeting to cancer cells via advanced EPR. Similar results were obtained with the comparison of Au_25_(SG)_18_ and larger AuZwMe_2_.^61,62^

**Figure 9:**
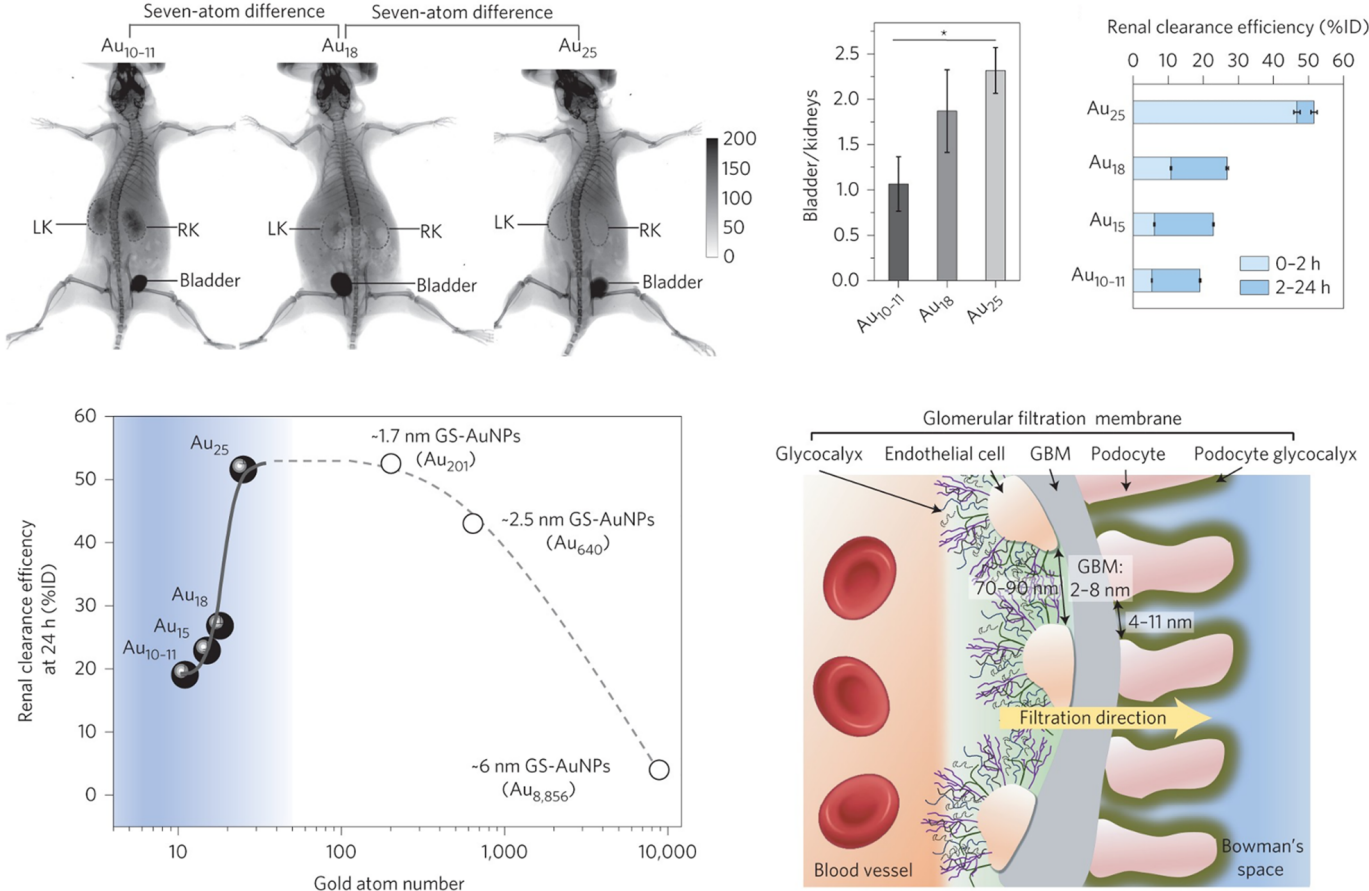
Whole-body X-ray images of mice, X-ray intensity bladder-to-kidney ratios, and renal clearance efficiency of intravenously injecting Au NCs at 40 min p.i., as well as the compositions of glomerular filtration membrane. Note: LK, left kidney; RK, right kidney. Reproduced from ref 60. Copyright 2017 Springer Nature Ltd.

## 4 Conclusions

In this Review, we mainly survey the syntheses and biomedical applications of water-soluble Au NCs. Most of synthetic methods can achieve large-scale yield and high purity. In particular, atomically precise Au_25_ NCs protected by the water-soluble SG, 11-MUA, *m*-MBA, *p*-MBA, MHA, Capt, BSA, and Cys ligands have been prepared (Figure 10) and exhibit outstanding advantages in bioimaging, biosensing, and biotherapy, especially for exploring the structure–property correlations for the ABME and PK of Au NCs. Nonetheless, there exist some challenges to synthesize more water-soluble Au NCs and further enhance the applications in biomedicine.

**Figure 10:**
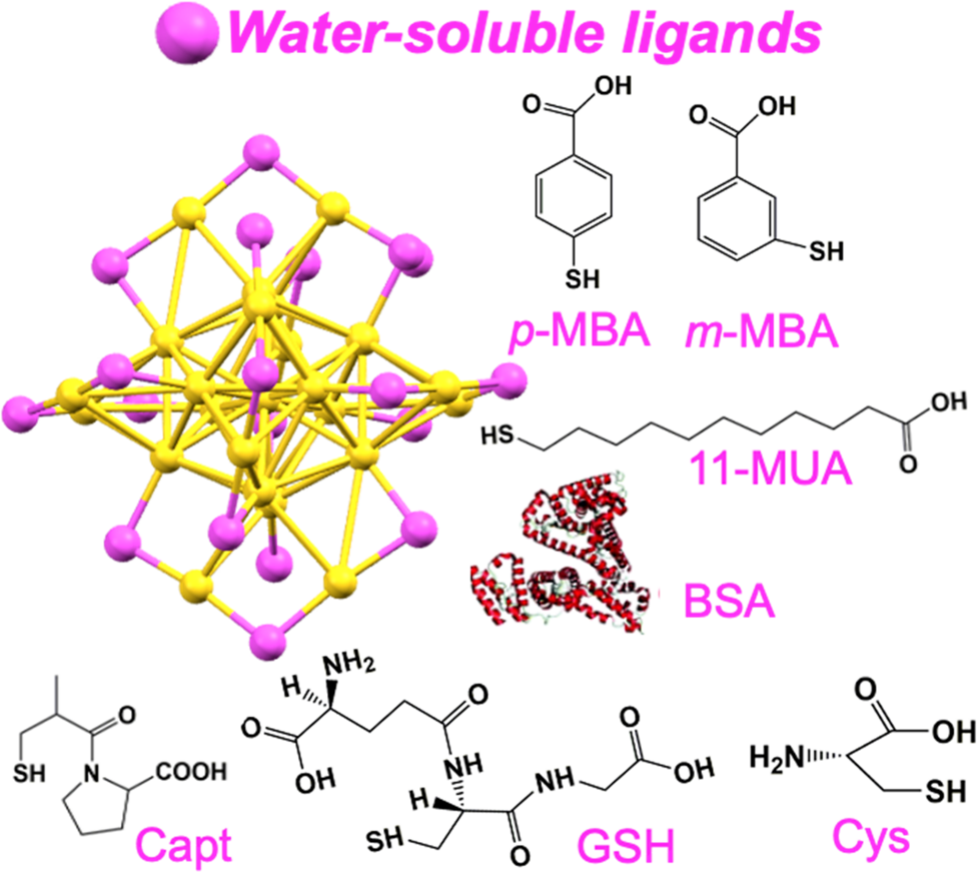
Water-soluble Au_25_ NCs protected by different ligands.

### 4.1 Synthetic Method and Structure Aspects

To date, only a few water-soluble Au NCs, such as Au_10–12_, Au_15_, Au_18_, and Au_25_, have been prepared at a large-scale. Meanwhile, the well-defined crystalline structure of water-soluble Au NCs has not been resolved because crystallization is difficult. Although we believe the bonding modes and crystalline structure of water-soluble Au_25_ to be similar to that of the hydrophobic Au_25_, the large steric ligands such as BSA stabilized Au_25_ NCs might possess a different Au-ligand binding mode and total crystal structure. For instance, BSA-Au_25_ almost has no absorption as shown by UV–vis spectra; namely, BSA-Au_25_ exhibits the different UV–vis spectra compared with the other water-soluble ligand (e.g., SG, 11-MUA, *m*-MBA, *p*-MBA, Capt, Cys) protected Au_25_ NCs, which further proved that there exist many differences in terms of the Au–ligand interaction and the optical effect from the BSA ligand with large steric hindrance. Therefore, exploring synthetic methods and the crystallization technology of water-soluble Au NCs for effectively determining the crystalline structures and synthesizing more water-soluble Au NCs with diverse compositions and structures are urgently desired, and play a significant role in fundamental studies. Furthermore, the key intermediate species formed in the synthetic process and the evolution of Au NCs compositions need to be monitored by utilizing in situ characterization tools, which can provide the reaction mechanism to further guide the design and synthesis of more Au NCs. In addition, machine learning can optimize the synthesis process and guide the experimental design, but we have to make a concerted effort to generate more data before training more machine learning models.

### 4.2 Biomedical Application Aspects

The size, length, charge, composition, and quantity of these ligands on the surface of water-soluble Au NCs can influence the optical properties, biodistribution, renal elimination, tumor consuming, and biodegradability of water-soluble Au NCs. Thus, carefully exploring the parameter effects on the biomedical properties is of significance for designing Au NCs for utilization in biomedicine. Moreover, Au NCs have been utilized for in vivo bioimaging for animal experiments. Nonetheless, in comparison with the developed luminescent materials, the QYs of Au NCs are relatively low, and are largely insufficient (Table 2, QYs < 20%). Therefore, the QYs and the luminescent property of Au NCs still need to be improved. Likewise, it is essential to further study the relationships among the structure, stability, and PL of Au NCs. In this regard, developing some new technologies to deeply analyze the biological process is greatly meaningful. Last but not least, we believe that the well-defined water-soluble Au NCs are not just about cancer treatment, but can also be utilized for the diagnosis of other diseases, for example, renal dysfunction^63–65^ and Alzheimer’s disease.^66,67^

**Table 2:** Luminescent Properties of Au NCs^a^

Au NCs	Em (nm)	FL	QYs (%)	ref
Au_18_(SG)_15_	745	1.6 μs	5.3	^68^
Au_22_ (SG)_16_	720	NA	<0.5	^69^
Au_22_ (SG)_17_				
Au_22_ (SG)_18_	665	1.37 μs	8	
Au_25_(SG)_18_	700	1.6 μs	0.19	^70^
BSA-Au_25_	640		6	^40^
Au_11_(SCH_2_CH_2_COO^–^)_7_(TOA^+^)_7_	417	NA	8.6	^71^

a Em = emission wavelength; FL = fluorescence lifetimes.

Apparently, the water-soluble Au NCs offer an ideal platform to develop some functional nanomaterials to solve human health problems. Using the precisely structured water-soluble Au NCs for biological diagnosis and therapy is just the beginning. Once the pharmacokinetic and PL properties, accompanied by the manifested therapeutic potential, are obviously proved at the preclinical level, we believe that these water-soluble Au-NCs can be rapidly utilized in clinical tests.
